# The effect of medical material management system app on nursing workload and stress

**DOI:** 10.1186/s12912-022-00806-4

**Published:** 2022-01-17

**Authors:** Yi-Tsao Chen, Yi-Cheng Chiu, Meng-Lan Teng, Pei-Hung Liao

**Affiliations:** 1 College of Nursing, National Taipei University of Nursing and Health Sciences, No. 365, Ming-te Road, Peitou District, Taipei City, 112 Taiwan; 2 Department of Nursing, National Taiwan University Hospital Hsin-Chu Branch, No.25, Lane 442, Sec.1, Jingguo Rd., Hsinchu City, 300 Taiwan; 3 School of Nursing, National Taipei University of Nursing and Health Sciences, No. 365, Ming-te Road, Peitou District, Taipei City, 112 Taiwan

**Keywords:** Nursing shift, Medical material management, Nursing workload, Work stress

## Abstract

**Aims:**

To develop a clinical medical material management App for nurses, in order to reduce their workload and improve the efficacy of medical material management.

**Design:**

The single-group pre- and post-test experimental design was adopted.

**Methods:**

The subjects were nurses in the intensive care units of a regional hospital in Hsinchu City enrolled by purposive sampling. Single-group pre-tests and post-tests were conducted. The research period was from November 2019 to March 2020. The workload, stress, and information acceptance of 57 nurses before and after the intervention of the Medical Equipment App were collected. The research tools included a structured questionnaire, which includes open questions that cover the aspects of workload, stress, and information acceptance intention of nurses, as well as a demographic questionnaire, which collects the basic personal data, including gender, age, years of service, educational level, nursing ability level, use ability of IT products, and unit type. The results were analyzed and compared using SPSS, APP Inventor, and data mining modeling to determine the effects of the App.

**Results:**

After employing the Shift Check App, the average workload of nurses was effectively reduced, in particular, the workload reduction of the N1 level nursing ability was greater than that of N2. In addition to satisfaction, the scores of information acceptance intention in all aspects, including behavioral intention, technology use intention, and contributing factors, all increased.

**Conclusion:**

The use of information technology products to assist medical material management in clinical practice has a significant effect on the load reduction of nurses and improvement of satisfaction.

**Clinical relevance:**

The App developed in this study can improve nurses’ work satisfaction, quality of care and workload reduction.

## Background

Nurses’ job duties include direct and indirect patient care, and they work under high work stress and heavy workload. Many studies have found a significant correlation between excessive nursing workload, burnout, and the lack of sense of accomplishment [1]. Nurses work in environments with heavy task loads, complicated interpersonal relationships, and changeable patient conditions; they also have to manage medical materials and other materials needed for medical care. The inventory management of medical materials affects the quality of care, and correct and efficient inventory management can ensure safe and reliable medical services [2]. Medical supplies routine count is an important pre-work in nursing activities. There are different routine count checklists according to the classification of medical materials, and the person responsible for the routine count is appointed by the team leader of each shift. The nurses need to check and register the items before the shifts start and confirm the quantity of materials and disinfection status. If the team leader of the next shift is unable to locate some materials, he/she needs to inquire the nurses of the previous shift about the location of the materials. The shift cannot be finished until the correctness of the medical materials is confirmed. The routine count of medical supplies generally takes about 15 min for each shift, which delays the shift handover time [3]. The purpose of this study was to develop an App suitable for nurses to manage medical equipment and materials, in order to reduce the nursing workloads and the shift handover.

### Nursing workload and stress

The factors affecting nursing workload include competency, work intensity, turnover rate, daily average hours of care, skill mix, and the ratio of registered nurses to average patient census. Nursing workload also affects patient outcomes, such as fall rate, bedsores, urinary tract infection, nosocomial infection, and patient satisfaction [4]. According to a literature review on nurses’ work stress in Australia, the sources of nurses’ stress include working overtime, role conflict, seeking support in response to strategies, seeking solutions to problems, and self-adjustment [5].. The same work stress factors are also found among nurses in Taiwan, such as overwork, sense of powerlessness, and work-family conflict [6]. The Taiwan Hospital Nursing Staff Stress Scale was used in previous studies to explore the correlation between the work stress, work burnout, and physical and mental health of clinical nurses [7], as well as the relationship between labor input during partial working hours and the work stress of nurses [8]. In 1996, Tsai and Chen [9] developed the Taiwan Hospital Nursing Staff Stress Scale to investigate the burden on nursing work caused by the duty to manage medical equipment [9].

The Nursing Activities Score (NAS) scale, which was developed by Miranda et al. in 2003 [10], covers seven aspects, and has been used to evaluate the nursing workload in an intensive care center (ICU) environment [10, 11]. Nasirizad et al. applied the NAS and NASA-TLX (NASA Task Load Index) to evaluate the physical and mental workload of nurses, and found that physical and mental workloads are statistically significantly correlated [12]. Other research on the factors affecting nursing care and nursing workload in ICUs also indicated that the workload of ICU nurses is affected by the personal characteristics of the nurses and the patients. For example, there is a direct correlation among female nurses caring for male patients, the increase of patients receiving nursing care and the extension of ICU stays, and the increase in the NAS [12].

### Applications for Mobile health care

Mobile Health App is a mobile application that conforms to the definition of a medical device by the U.S. Food and Drug Administration and can be used as a regulated medical device accessory or as a mobile platform for conversion into a regulated medical device [13]. In the M2M (machine to machine) mode, medical data are uploaded to the App via the Internet, Bluetooth, radio-frequency identification (RFID), and other technologies, while the medical staff can make medical judgments according to the recorded data [14, 15]. This App can provide assistance in the aspects of care tasks to reduce the nursing workload and improve the quality of care. In 2016, Chiang explored nurses’ experiences of using smartphone Apps to provide assistance in home care for patients with chronic diseases, and found that the Apps could help nurses to make timely decisions on patients’ conditions, and promote nurse-patient relationships. However, in consideration of information security, Chiang also suggested that it is necessary to raise nurses’ awareness on information security when using mobile care in the future [16]. Another study on the availability of electronic health records and information technology capabilities also showed that the unfriendly design of electronic medical records is the main source of pressure and psychological distress for nurses [17]. Hence, to provide a user-friendly electronic medical record system and tools for the medical insurances, medical institutions should strengthen the tool design and provide education and training for nurses [17].

### Information system effectiveness and information acceptance intention assessment

**Fig. 1:**
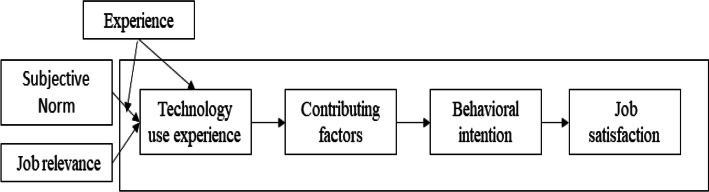
Information system acceptance mode

Venkatesh and Davis [18] extended the technology acceptance model (TAM) and proposed TAM 2, which considers that social influence and cognitive instrumentality affect perceived usefulness. Specifically, subjective norms, images, job relevance, output quality, and result demonstrability can all affect perceived usefulness, thus, use intention is affected by subjective norms. Usage experience and voluntariness are the interference variables of use intention, meaning that subjective norms significantly affect the users’ usage intention when the users lack experience or use a system involuntarily. Thus, user inexperience significantly affects perceived usefulness. Based on the TAM and the above discussions, information acceptance intention is users’ intention to use the technology, such as the reliability, convenience, and effectiveness of the system. The contributing factors of external support assistance affect the users’ behavioral intention to use the technology, which subsequently affects job satisfaction. The information system acceptance mode was developed, and it includes the four aspects of technology use experience, contributing factors, behavioral intention, and job satisfaction (Fig. 1) [19, 20]. The Cronbach’s α of internal consistency reliability are all greater than 0.8, and thus, can effectively distinguish relevant intentions of an information application.

### Text mining

Text mining technology has been developed from the use of book classification to the information extraction of unstructured text [21]. During the process of editing, organizing, and analyzing, text mining can discover hidden features, correlations, and novel and interesting patterns that provide decision-makers with specific information [22]. The main functions of text mining include data retrieval and processing, word segmentation, feature selection, classification and clustering, and text representation and interpretation [23]. Text mining can present unstructured text data through data visualization, and a “word cloud” can be developed [24, 25], which is a cloud-like figure composed of various words and expressions. Natural language processing technology has obvious limitations [26]; therefore, the new generation of technology has been committed to deep discovery, with an attempt to distinguish the implicit meanings of idioms, sarcasm, and innuendo [21]. Recent studies in Taiwan have established sentiment analysis [27]. The semantic differential technique, which divides words into two corresponding adjectives, such as good and bad, strong and weak, passive and active, was developed by Snider and Osgood in 1969 [28]. The semantic differential technique uses the word cloud analysis to quickly extract the important words of an article, applies the correlation analysis to determine the important words related to the topic, employs the context analysis to decompose the important context of the article, employs the cluster analysis to classify the key words of the article, and conducts the emotional analysis to discuss the content of public opinion according to positive and negative sides. Finally, a complete set of text exploration and analysis was formed with the aid of visualization [29].

## Methodology

### Research design

The research design of this study was single-group pre- and post-test. The handheld nursing shift handover application (Shift Check App) was provided to nurses in four ICUs. The experiment was conducted in a clinical setting at a regional teaching hospital in northern Taiwan from September 1, 2019 to March 31, 2020. The “Nursing Workload Visual Analogue Scale (VAS)” and “Taiwan Hospital Nursing Staff Stress Scale” were employed to collect the participants’ data before and after the intervention (after three months of using the App) in order to measure their workload and stress in the previous week. The scale on “information acceptance intention” was used to explore the influence of the intervention of the App on nurses.

This study was submitted to and approved by the Institutional Review Board (IRB No.: 107–56-E) of the National Taiwan University Hospital Hsin-Chu Branch. All participants were required to read the pre-participation instructions and sign the written consents prior to the study. To maintain data confidentiality and privacy, the questionnaires were distributed and collected individually in envelopes after being sealed. The questionnaires were filled out by the nurses themselves anonymously, and a designated box was placed in the nursing station for the nurses to return the questionnaires independently. The researchers collected the questionnaires, and strictly observed the principle of confidentiality.

### Research objects

The estimated sample size was analyzed by statistical software (SPSS 22.0/GPower3.1), and the estimation method was based on three main independent variables, with the α value of 0.05, power of 0.8, and effect size of 0.3. Hence, a single group required to be at least 70 nurses for the sample to be representative, and this study collected data according to the units that met the sample selection conditions.

The participants were purposefully sampled from ICU nurses of a regional hospital in Hsinchu City, and single-group pre-tests and post-tests were conducted. Workload, stress, and information acceptance of 70 nurses were measured before the intervention of the Shift Check App and measured again three months after the intervention. The differences before and after the intervention were then compared.

The inclusion criteria included: (1) nurses that have worked in the hospital for six months and taken the department shift for six months; (2) nurses that were willing to use the Shift Check App; (3) nurses that agreed to complete the anonymous pre-test and post-test questionnaires. The exclusion criteria were: (1) non-clinical users and head nurses; (2) lost trackers, who did not complete the questionnaire or had personnel changes.

### Research tools

The questionnaire adopted in this study consists four parts:

1. Basic personal data: The data include gender, age, years of service, educational level, nursing ability level, use ability of IT products, and unit type.
2. Nurse Workload Scale: In terms of factors related to nurses’ job satisfaction, studies have found that nursing workload is significantly and negatively correlated with job satisfaction, that is, the heavier the workload, the worse the job satisfaction [30]. This study referred to Tsai and Chen’s “Taiwan Hospital Nursing Staff Stress Scale” [9], which was modified from Benoliel et al. [31]. Tsai and Chen’s scale [9] has Cronbach’s α of above 0.84, and is measured based on a 10-point score line, where 1 denotes the lowest workload and 10 denotes the greatest workload.
3. Nursing Staff Stress Scale: This study adopted Tsai and Chen’s “Taiwan Hospital Nursing Staff Stress Scale” [9], which was modified from Benoliel et al. [31]. This scale has four stress dimensions, including “personal response”, “work concern”, “work competence”, and “inability to complete personal work”, with 47 items in total. The measurement is based on a 9-point Likert scale, ranging from 0 (the lowest stress) to 9 (the greatest stress). Cronbach’s α of each dimension is above 0.84. Items 2, 3, 4, 7, 8, 9, 11, 12, 13, 14, 15, 16, 17, 19, 23, 24 and 25 in the second section of the scale are related to the dimension of personal response; items 1 to 12 in the first section and item 1 in the second section are related to the dimension of work concern; items 5, 6, 10, 18, 20, 21, 22, 30, 31, 33, and 34 in the second section are related to the dimension of work competence; items 26, 27, 28, 29, 32, and 35 in the second section are related to the dimension of inability to complete personal work. There are 11 reverse questions, namely item 5, 6, 10, 18, 20, 21, 22, 30, 31, 33, and 34 in the second section.
4. Information System Acceptance Mode: This study referred to TAM 2 of Venkatesh and Davis [13–15], and discussed with scholars and experts in the related field. The content includes 22 items in four dimensions, namely behavioral intention, contributing factors, technology use intention, and job satisfaction. A 5-point Likert scale is used, ranging from 5 (strongly agree) to 1 (strongly disagree). In terms of expert validity, three experts (a nursing information expert, a nursing clinical expert, and an information engineer) were invited to verify the appropriateness and clarity of the questionnaire contents. The total content validity index is 0.90, the Cronbach’s α of internal consistency for the scale is greater than 0.9, and the values of Cronbach’s α of internal consistency reliability for the four dimensions are all greater than 0.9. Thus, this scale could effectively distinguish the relevant intentions of information application.

**Fig. 2:**
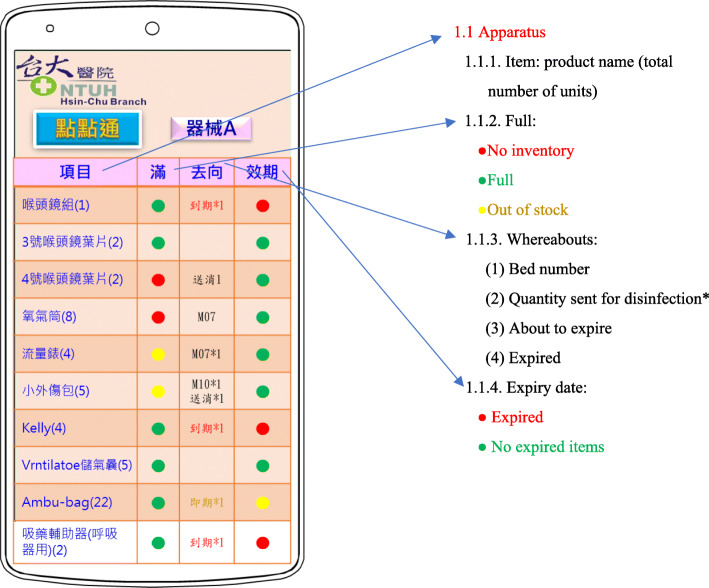
Shift APP function diagram

This study developed the Shift Check App according to user experiment, design thinking, and the needs of clinical nurses. The researchers observed the working processes of the nurses’ shifts and the disinfection procedures, interviewed nurses and supply room operators on their work needs, planned warehousing and delivery procedures of the supply room, confirmed the validity period of disinfected articles, and reviewed other major tasks that are relevant to the supply room operation, a convenient summary table was created to facilitate the cross-departmental use. The design team consisted of nursing information experts, nursing clinical professionals, and information engineers. Android Studio 3.5.2 was used to design the editor developments, and the PHP 5.6.32 (Personal Homepage Program, a programming language) command code was used to access the correlation database MySQL DB 5.6.41 (it is a multi-user, multi-execution sequence SQL database server, which can effectively arrange, file, and tabulate database software for more efficient query and sorting), and transmit and receive data (Fig. 2).

### Data processing and analysis

The collected data were analyzed using SPSS 25.0 statistical software, and statistical methods included percentage, mean, Pearson correlation coefficient, paired t test, independent-samples t-test, and analysis of variance (ANOVA).

## Result

### Basic demographic variables and correlations of the participants

The mean age of the participants is 29.75 ± 6.99 years old. There are 10 nurses from RCC (Respiratory Care Center) (17.5%), 11 from MICU (Medical Intensive Care Unit) (19.3%), 25 from CCU (Cardiac Intensive Care Unit) (43.9%), and 11 from SICU (Surgical Intensive Care Unit) (19.3%). The nursing competency levels are: N: 13 (22.8%), N1: 168 (14.0%), N2: 29 (50.9%), N3: 6 (10.5%), and N4: 1 (1.8%). Pearson correlation coefficient analysis shows that age is negatively correlated with perceived ability to use IT products, indicating that older nurses perceive more difficulty operating the App (r = − 0.435). The workload variable was analyzed by quartile range. The quartile analysis is the symmetrical distribution data (the median must be equal to the arithmetic mean of the third quartile and the first quartile). The half for the quartile deviation is equal to the median absolute deviation (MAD) and the median reflects the concentration trend.

$$ \mathrm{Equation}:\mathrm{IQR}=\mathrm{Q}3-\mathrm{Q}1 $$

**Fig. 3:**
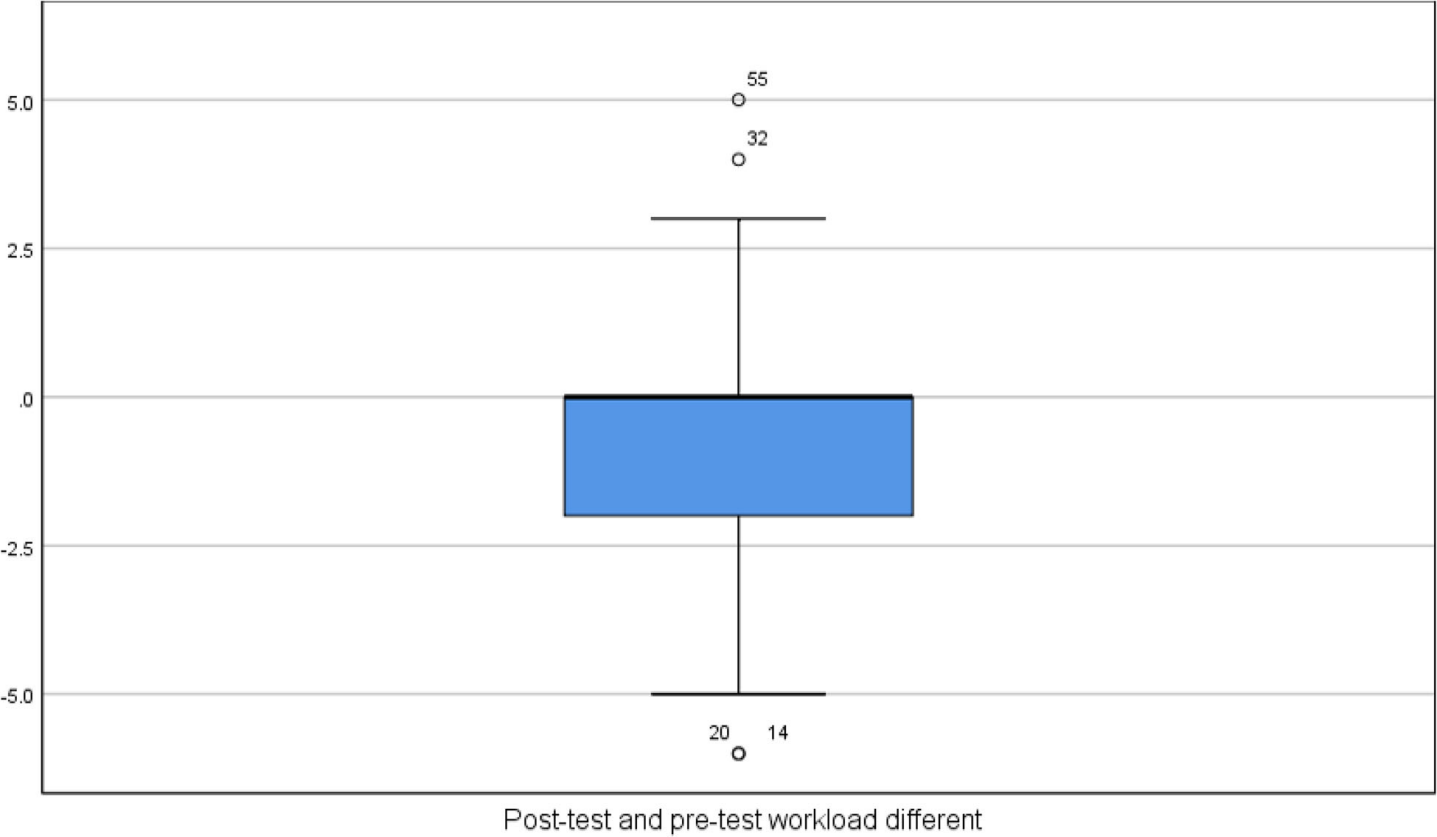
Post-test and Pre test workload different Boxplot

From the post-test and pre-test workload Stem-and-Leaf Plot and box plot, we can find that the pre-test load is mostly moderate and heavy, and the post-test load is mainly light and medium (Tables 1, 2, Fig. 3)

**Table 1:** Descriptive statistical analysis of demographic data of the participants (*N* = 57)

Category change (***N*** = 57)	Mean ± SD	Number of people (%)
Age * years old	29.75 ± 6.99	
**Annual salary (including that beyond hospital), /month**	94.39 ± 80.67	
≦24		10 (17.5%)
25 ~ 48		11 (19.3%)
49 ~ 72		7 (12.3%)
73 ~ 96		12 (21.1%)
> 96		17 (29.8%)
**Gender**		
Female		52 (91.2%)
Male		5 (8.8%)
**Educational level**		
Junior college		14 (24.6%)
Bachelor		43 (75.4%)
Master or above		0 (0%)
**Ward**		
Internal Medicine Department		21 (36.8%)
Surgery Department		36 (63.2%)
**Working unit**		
RCC		10 (17.5%)
MICU		11 (19.3%)
CCU		25 (43.9%)
SICU		11 (19.3%)
**Advanced nursing ability**		
N		13 (22.8%)
N1		8 (14.0%)
N2		29 (50.9%)
N3		6 (10.5%)
N4		1 (1.8%)
**Self-perceived competency of 3C equipment**	3.39 ± 0.73	
Very bad (1 point)		2 (3.5%)
Poor (2 points)		1 (1.8%)
Normal (3 points)		28 (49.1%)
Good (4 points)		25 (43.9%)
Very good (5 points)		1 (1.8%)

**Table 2:** Workload analysis results before and after the Shift Check App intervention (*N* = 57)

Effectiveness Indicator	Before Intervention (*N* = 57)		After Intervention (*N* = 57)		*T*^*a*^	*P*-value
	Mean ± SD	Number (%)	Mean ± SD	Number (%)		
Workload	4.49 ± 2.05		3.82 ± 1.68		2.21	.031*
No		3 (5.3%)		2 (3.5%)		
Mild		14 (24.6%)		19 (33.3%)		
Moderate		32 (56.1%)		34 (59.6%)		
heavy		8 (14.0%)		2 (3.5%)		

*. *P* < .05

^*a.*^*Paired t test*

### Impact of shift check app on nursing workload and stress

The average nursing workload was 4.49 ± 2.05 points before using the Shift Check App, and 3.82 ± 1.68 points after using the App, suggesting that the average workload reduced after using the App. Comparison of nurses’ stress before and after using the App shows that in the personal response dimension, the average score is 2.6 ± 1.50 points for pre-test, and 2.72 ± 1.48 points for post-test; in the work concern dimension, the average score is 2.53 ± 1.36 points for pre-test, and 2.56 ± 1.18 points for post-test; in the work competence dimension, the average score is 3.72 ± 1.39 points for pre-test, and 3.65 ± 1.38 points for post-test; in the inability to complete personal work dimension, the average score is 3.46 ± 1.48 points for pre-test, and 3.25 ± 1.43 points for post-test. In the four dimensions of the stress scale, the post-test scores of personal response and work concern were higher than the pre-test scores, indicating a slight increase in the stress of these two dimensions. The post-test scores of work competence and inability to complete personal work were lower than the pre-test scores, indicating a slight decrease in the stress of these two dimensions. However, there was no statistically significant difference in the scores of each dimension before or after the intervention (all *p* > .05). According to the results, among the four dimensions of the stress of nursing staves, the scores of personal response and work concern in post-tests were higher than those in pre-tests, indicating that the pressure of the two dimensions increased slightly. The scores of work competence and inability to complete personal work in post-tests were lower than those in pre-tests, suggesting that the stress of these two dimensions decreased slightly. However, there was no statistically significant difference between the scores of each dimension before and after the intervention (Table 3).

**Table 3:** Analysis results of stress scale before and after the intervention of the Shift Check App (*N* = 57)

Effect indicator	Pre-test (***N*** = 57)	Post-test (***N*** = 57)	T^*a*^	***P***-value
	Mean ± SD	Mean ± SD		
**Stress of nurses**				
Personal response			−0.184	.855
Total score	45.37 ± 25.58	46.18 ± 25.16		
Grand average	2.6˙ ± 1.50	2.72 ± 1.48		
Work concern			−0.147	.884
Total score	32.85 ± 17.71	33.28 ± 15.35		
Grand average	2.53 ± 1.36	2.56 ± 1.18		
Work competence			0.334	.740
Total score	40.95 ± 15.27	40.12 ± 15.22		
Grand average	3.72 ± 1.39	3.65 ± 1.38		
Inability to complete personal work			0.899	.372
Total score	20.79 ± 8.84	19.51 ± 8.58		
Grand average	3.46 ± 1.48	3.25 ± 1.43		

*. *P* < .05

^*a.*^*Paired t test*

### Nursing workload and stress

Based on the variables, such as nursing ability, educational level, and ward characteristics, independent sample t test was conducted to explore the correlation differences of nursing workload and stress before and after using the Shift Check App in each variable group. ANOVA was used to analyze the nursing workload and stress in four wards, in order to determine the correlation difference before and after the intervention of the Shift Check App.

1. The nursing ability was divided into N1 and below and N2 and above (clinical N2 nurses have completed PGY2 (post graduate year two of complete basic training); the nursing workload variance before and after was above N1, and the workload was lower than N2, reaching statistically significant difference. This suggests that compared with the groups above N2 (including N2), the workload degree of the groups below N1 (including N1) decreased after the intervention of the Shift Check App. In terms of the dimension of work concern of nurses’ stress, there was statistically significant difference before and after the intervention, indicating that the work concern of nurses’ stress of the groups below N1 (including N1) decreased more after the intervention of the Shift Check App than that of the groups above N2 (including N2), while the stress of the groups above N2 (including N2) was higher after the intervention.
2. ANOVA was used to calculate the difference scores of the nursing workload and stress in the four wards before and after the intervention of the Shift Check App. There were statistically significant differences in the work concerns of the nurses’ stress in different wards. According to Scheffe test, the work concern difference score of MICU was greater than that of SICU, indicating that the decrease of nursing stress in MICU after the intervention of the Shift Check App was greater than that in SICU, reaching a statistically significant difference.

### Difference of nurses in information system acceptance intention after using the shift check app

Behavioral intention was analyzed by paired t test, and the average score was 3.19 ± 0.77 points for the pre-test, and 3.46 ± 0.78 points for the post-test. The average behavioral intention score of the post-test was higher than that of the pre-test. As for technology use intention, the average score was 3.10 ± 0.63 points for the pre-test, and 3.43 ± 0.65 points for the post-test. The average technology use intention score of the post-test was higher than that of the pre-test. As for contributing factors, the average score was 3.20 ± 0.44 points for the pre-test, and 3.64 ± 0.92 points for the post-test. The average contributing factors score of the post-test was higher that of the pre-test. In terms of job satisfaction, the average score was 3.37 ± 0.50 points for the pre-test, and 3.43 ± 0.46 points for the post-test, which does not reach statistically significant difference. As shown in Table 2, the scores of each dimension of information acceptance intention increased after the intervention. With the exception of satisfaction, the factors of behavioral intention, technology use intention, and contributing factors all show statistically significant difference. The results prove that the intervention of the Shift Check App improved the information acceptance intention of users (Table 4).

**Table 4:** Analysis results of information acceptance intention before and after the Shift Check App intervention (*N* = 57)

Effectiveness Indicator	Before Intervention (*N* = 57)	After Intervention (*N* = 57)	*T*^*a*^	*P*-value
	Mean ± SD	Mean ± SD		
Behavioral intention			−2.05	.045*
	Total average	3.19 ± 0.77	3.46 ± 0.78	
Technology use intention			−3.32	.002*
Total average	3.10 ± 0.63	3.43 ± 0.65		
Contributing factors			−3.67	.001*
Total average	3.20 ± 0.44	3.64 ± 0.92		
Job satisfaction			−0.73	.468
Total average	3.37 ± 0.50	3.43 ± 0.46		

*. *P* < .05

^*a.*^*Paired t test*

### Qualitative feedback from users after using the shift check app

The open data collected in the questionnaire was explored in the form of text mining and presented in the form of a word cloud. It was found that “waste” and “not environmentally-friendly” were selected before the intervention of the Shift Check App, and “convenience” was selected after the intervention. Then, the keywords presented by the word cloud were analyzed by public opinion. According to the analysis of public opinion by Academia Sinica, when the advantages of the Shift Check App were analyzed as “convenience”, the result was positive emotion. When the disadvantages of the Shift Check App were analyzed as “waste”, the result was negative emotion.

## Discussion

### Comparison of basic data of participants with other similar studies

The average age of nurses participating in this study was 29.75 ± 6.99 years old, and older nurses perceived their ability to use IT products to be lower (r = −.435). In research on the degree of acceptance of science and technology, demographic data, such as age, education level, working seniority, work unit, and rank, are often used to discuss whether they would affect the willingness or behavior of using technology. For example, relevant studies have been conducted to compare the satisfaction of nurses in different ICUs (internal medicine and surgical intensive care units) regarding the use of clinical information systems [32]. There have also been comparisons of the differences in the age, seniority, and rank of nurses using or not using mobile nursing stations [33]. Those studies found that there is a significant difference between age and the factors related to the acceptance model of information use technology (F = 5.018). According to the Scheffe test, people under 30 years old (M = 81.48 ± 13.16) are more sensitive to cognitive usefulness than those aged 31–40 years (M = 73.82 ± 12.66) and 41–50 years (M = 81.48 ± 13.16), indicating that younger people are more likely than older people to agree that an information system is useful [34]. Another study pointed out that nurses at the N level have more positive acceptance of nursing information usage than nurses at the N1 and N2 levels (F = 3.95). The results of this study are consistent with the above results, which is because the younger groups have more time and opportunities to use IT products, and thus, have better adaptability and usage ability when learning new IT products. Some correlation studies [35, 36] suggested that nursing information ability is better for older nurses in N4 level, more than 4 h (including) of online learning experience, and more than 4 h (including) of online access per week, which is different from the results of this study. This difference may be related to the provision of sufficient nursing information training in the unit.

### Results of nursing workload and stress after the intervention of the shift check app

Among the 44 common work stressors identified in this study regarding nursing workload and work stress, the top three items are: “all kinds of assessment preparation work must be prepared by writing various documents or reports”, “when a property is lost during a shift, nurses have to pay for it themselves”, and “participation in more complex surgery”. Reducing the loss of property during a shift-handover check can reduce the work stress of nurses [37]. In a study of 121 male nurses, Hsu et al. found that work stress, including workload, role conflict, and workplace interactions, could explain 45.8% of the variance in workplace fatigue [38]. Lu et al. found that administrative staff should regularly examine nurses on possible high work stress and overload, understand the causes of their overload, simplify their workload, and re-divide the workload, which can help to reduce the work stress [39]. In conclusion, the relief of nursing work stress in the above studies depends on the simplification of the working process. According to the above studies, after the intervention of information applications, work convenience is increased, and the nursing workload is effectively reduced to lower their work stress, which is consistent with the results of this study.

### Discussion on the nurses’ information acceptance intention after the intervention of the shift check app

The results of this study show that after the intervention of the Shift Check App, there were significant differences in the information acceptance intention of nurses in the three dimensions of behavioral intention, technology use intention, and contributing factors. Other research results show that the acceptance degree of an information system is the highest in terms of perceived usefulness, followed by perceptual ease of use, which is consistent with this study [40]. However, information equipment is prone to reduced willingness to use due to poor hardware equipment, screen switching, storage, delay, crash, or difficulties in using system functions, which cannot meet the needs of users [41, 42]. Therefore, a convenient and stable technology system combined with strong external support capability can improve users’ behavioral intention to change, enhance their willingness to use, and increase job satisfaction. In this study, the application process of the Shift Check App could not be promoted by the entire hospital, along with the malfunctioning and signal transmission problems, thus, the effect on satisfaction was low, which is consistent with the research results of Li et al. [41].

### Research limitation

Although there were 70 participants at the beginning of this study, on January 16, 2020, the hospital adjusted its manpower allocation due to the impact of COVID-19, thus only 57 participants completed this study. Our future study will employ the Shift Check App in more units, collect the opinions of nurses from each unit, and optimize the system functions and user-friendly design of the App to reduce the nursing workload.

## Conclusion

According to the analysis results of this study, the intervention of the Shift Check App could effectively reduce the average workload and stress of nurses, and the workload of N1 nurses decreased more than that of N2 nurses. In terms of the work concern of nurses’ stress, the work stress of N1 nurses decreased more than that of N2 nurses. In terms of the work concern of nurses’ stress in different units, this study found that the work concerns of nurses in the MICU decreased more than those in the SICU after using the Shift Check App. With the exception of satisfaction, after the intervention, the scores of all aspects of the information system acceptance intention were increased. Therefore, for users, the intervention of the Shift Check App mostly improved information acceptance intention. This study also found that age is negatively correlated with the participants’ use ability of IT products, which indicates that older nurses find more difficulty in using IT products. In the qualitative feedback part, the word cloud and public opinion analysis showed the negative emotions of “waste” and “not environmentally-friendly” before the intervention, and a positive emotion clause of “convenience” after the intervention.

## Data Availability

The data that support the findings of this study are available from the corresponding author, upon reasonable request.

## Abbreviations

VAS: visual analogue scale
RFID: radio-frequency identification
TAM: technology acceptance model
APP: application
ANOVA: analysis of variance
